# Laparoscopic Assisted Vaginal Hysterectomy, Setting Up a Service at a Peripheral Teaching Hospital

**DOI:** 10.1155/DTE.3.121

**Published:** 1996

**Authors:** Jim Tsaltas, Gab Kovacs, Jenny Dennis, Amanda Pratt

**Affiliations:** 1 Department of Obstetrics and Gynaecology Monash University Monash Medical Centre and Box Hill Hospital Melbourne Australia; 2 Department of O & G. Monash Medical Centre 246 Clayton Road Clayton 3168 Australia

## Abstract

The establishment of a laparoscopically assisted hysterectomy program at Box Hill Hospital is described. The first eight cases have been reviewed and recommendations are made to other gynaecology units who wish to establish a minimally invasive gynaecological surgery unit.


*Diagnostic and Therapeutic Endoscopy*, 1996, Vol. 3, pp. 121–124
Reprints available directly from the publisher
Photocopying permitted by license only

© 1996 OPA (Overseas Publishers Association)
Amsterdam B.V. Published in The Netherlands
by Harwood Academic Publishers
Printed in Malaysia

### Laparoscopic Assisted Vaginal Hysterectomy, Setting Up a Service at a Peripheral Teaching Hospital

JIM TSALTAS[1,*], GAB KOVACS[2], JENNY DENNIS[3], and AMANDA PRATT[4]

*Department of Obstetrics and Gynaecology, Monash University, Monash Medical Centre and Box Hill Hospital, Melbourne*

*(Received 9 August 1995; Revised 8 May 1996; In final form 24 June 1996)*

**The establishment of a laparoscopically assisted hysterectomy program at Box Hill Hospital is described. The first eight cases have been reviewed and recommendations are made to other gynaecology units who wish to establish a minimally invasive gynaecological surgery unit.**

*Keywords:* Laparoscopic assisted vaginal hysterectomy, training

### INTRODUCTION

The use of laparoscopy has been widespread in gynaecology during the past two decades. It has become evident that complex gynaecological procedures including hysterectomy can be performed using laparoscopic techniques [1]. By avoiding major abdominal incisions, hospital and associated costs are decreased and post operative pain and morbidity are reduced [2].

In 1989 Reich and DeCaprio reported on the first laparoscopic hysterectomy [1]. Since that time it has been performed at a number of centres around the world and several reports have been published [3,4,5,6,7,8,9,10,11].

Laparoscopic hysterectomy is becoming an acceptable alternative to abdominal hysterectomy when vaginal hysterectomy is considered difficult or inappropriate [8,10,11]. However as it is a new technique it will have to be learnt by gynaecologists who wish to use it. The Royal Australian College of Obstetricians and Gynaecologists has recommended guidelines for training clinicians in advanced laparoscopic techniques. This paper discusses the feasibility of such a training program in a small teaching hospital.

### METHODS

Box Hill Hospital is a teaching hospital of Monash University in Melbourne, Australia. It has an active and progressive department of obstetrics and gynaecology. The gynaecologists within this group saw the evolvement of advanced laparoscopic surgery and

[1]Gynaecologist, [2]Professor of Obstetrics and Gynaecology, [3]Charge Nurse, [4]Registered Nurse.
*Corresponding author. Tel.: +61-3-9550-5374. Fax: +61-3-9550-5389.
Present address: Department of O & G., Monash Medical Centre, 246 Clayton Road, Clayton, 3168 Australia.

were keen to commence an endoscopy service. Four of the surgeons, including the authors, attended courses and operated under supervision to develop skills in advanced laparoscopy and the number of procedures endoscopically steadily increased. This culminated in a desire to perform laparoscopic hysterectomy. The three surgeons had performed this procedure elsewhere but not at this hospital. The department was keen to provide this service but wanted to do it safely and to establish ongoing training service for the other gynaecologists in the unit who were interested in learning this new technique.

A working party was established between the medical staff and the operating theatre nursing staff to discuss laparoscopic hysterectomy. The nursing staff were already skilled in laparoscopic cholecystectomy as performed by the general surgeons. A thorough review was made of laparoscopic instruments that were already available and a list was drawn up of the instruments that would be required for laparoscopic hysterectomy. Two senior operating suite nurses were allocated to the gynaecological laparoscopic team. With respect to safety and training it was decided that these two sisters would always scrub for these cases. If other nursing sisters were keen to become involved they would have to scrub with one of the two senior sisters appointed to the service.

The Department of Obstetrics and Gynaecology based their training guidelines on the Royal Australian College of Obstetrics and Gynaecology and the Australian Gynaecological Endoscopy Society guidelines for training in advanced operative laparoscopy [12]. Initially it was felt safer to have two of the three experienced operative laparoscopists performing the laparoscopic hysterectomies with other gynaecologists being in theatre to observe and ask questions. Once everybody felt comfortable with this new technique, in particular, the senior sisters involved with the case, other gynaecologists from the department would be involved at a preceptorship level initially assisting and then acting as the chief surgeon. Under no circumstances was an inexperienced surgeon to perform the procedure on their own until the preceptors and the department of Obstetrics and Gynaecology were happy with their skills. The department felt that each surgeon should perform 5 procedures as the assistant prior to being the principal surgeon. At this point the preceptor would assist.

Of the eight women in this initial study (Table I), seven had menorrhagia, two with fibroids, and the eighth patient had CIN III. In none of these women was vaginal hysterectomy possible. The patients were aged from thirty six to forty eight (mean forty four years). The procedure was performed with the patients in Trendellenburg position. The ureter was routinely displayed by dissection. The infundibulo-pelvic and broad ligaments were taken with ENDO GIA thirty laparoscopic stapling device (Auto Suture Company, Adelaide, Australia). The uterine vessels were also taken laparoscopically either with bipolar diathermy or staples. The procedure was then completed vaginally. All pedicles were checked laparoscopically for haemostasis at the end of the procedure. The peritoneal cavity was irrigated with normal saline. The

TABLE I

| Age | Indications for LAVH | Procedure time (including anaesthesia) | Hopital stay | Complications |
|---|---|---|---|---|
| 36 | Menorrhagia | 120 min | 3 days | Nil |
| 44 | Menorrhagia | 105 min | 2 days | Nil |
| 45 | Menorrhagia | 95 min. | 3 days | Nil |
| 45 | CIN III | 180 min. | 4 days | Post Op Fever |
| 44 | Menorrhagia/fibroids | 150 min | 3 days | Nil |
| 43 | Menorrhagia/fibroids | 120 min | 3 days | Nil |
| 47 | Menorrhagia | 90 min | 2 days | Nil |
| 48 | Mild Endometriosis/ menorrhagia | 110 min | 3 days | Nil |

incisions were all sutured with 2.0 nylon. Neither a catheter nor a pack were inserted.

### RESULTS

The operating time, from induction of anaesthetic to the end of the procedure, for these patients ranged from ninety minutes to one hundred and eighty minutes (mean one hundred and twenty minutes). The time in hospital ranged from two to four days (mean of 2.87). The only complication was that of post operative fever which responded to antibiotics. A further statement about the significance of any complications cannot be made because of the small number of cases described here.

### DISCUSSION

Laparoscopic hysterectomy has and will continue to gain increasing attention from the gynaecological community. It is not only the gynaecologists who are becoming aware of this procedure but the media has publicised the advantages of minimally invasive surgery to a responsive consumer market. This has encouraged more and more gynaecologists to perform laparoscopic hysterectomy. Advanced laparoscopic surgery has also given peri-operative nurses an opportunity to expand their role. The use of a television monitor makes the procedure visible to all in the theatre. Procedures which were once difficult to view can be seen clearly, enhancing the understanding of the anatomy and the surgical techniques. As Garry *et al.*[7,13] believed that adequate training must be obtained by both the gynaecologist intent on performing laparoscopic hysterectomies and the theatre nurses who will be assisting them, the nurses who were involved in our unit were an integral part of our team. They took on new responsibilities such as actually assisting the surgeon, monitoring how the procedure was progressing, assuring the safety at all times during the procedure, preparing and checking equipment, being prepared and continually alert for a wide range of possible incidents such as equipment failure, breakages of fragile instruments and the ability to quickly troubleshoot as required. An experienced theatre nurse is essential to allow the procedure to run quickly and smoothly.

The gynaecologists must have a thorough knowledge of pelvic anatomy, and be familiar with available laparoscopic equipment and have an understanding of electro surgery and know each instrument's use and limitations. They should already be proficient in performing laparoscopic procedures on the tubes and ovaries. Finally a preceptorship with the surgeon experienced with laparoscopic hysterectomy is the most efficient and safe way to learn this new technique.

We are trying to incorporate all these principles into setting up a laparoscopic service at our hospital. In particular, each surgeon must train under a preceptor until the preceptor is happy to recommend that the surgeon can operate without supervision. This allows laparoscopic hysterectomy to be performed safely and to slowly be implemented into the clinical service of the hospital. The surgeon is fed back information regarding their progress by the preceptor and the chairman of the department.

We will stress to every hospital wishing to commence advanced laparoscopic surgery of all types and, in particular, laparoscopic hysterectomy that careful planning, discussion and co-operation with our nursing colleagues is essential. Each hospital should implement a training, accreditation, and quality assurance program so that they are satisfied their surgeons are performing this procedure safely and with an acceptable complication rate. Following these principles will enable all hospitals with interested gynaecologists to establish such a service.

### *References*

[1] Semm, K. (1976). *Endoscopic Abdominal Surgery*, Year Book Med. Publ. Chicago, p. 180–182.
[2] Levine, R. (1985). Economic impact of pelviscopic surgery. *Journal of Reproductive Medicine*, 30, 655–657.
[3] Liu, C. Y. (1992). Laparoscopic hysterectomy—a review of seventy two cases. *Journal of Reproductive Medicine*, 37, 351–354.
[4] Nezhat, F. *et al.* (1992). Laparoscopic versus abdominal hysterectomy. *Journal of Reproductive Medicine*, 37, 247–250.
[5] Phipps, J. H. *et al.* (1993). Comparison of laparoscopically assisted vaginal hysterectomy and bilateral salpingo-oophorectomy with conventional abdominal hysterectomy and salpingo-oophorectomy. *British Journal of Obstetrics and Gynaecology*, 100, 698–700.

[6] Raju, K. S. *et al.* (1994). A randomised perspective study of laparoscopic vaginal hysterectomy versus abdominal hysterectomy with bilateral salpingo-oophorectomy. *British Journal of Obstetrics and Gynaecology*, 101, 1068–1071.

[7] Wood, E. C. *et al.* (1992). Hysterectomy; a time of change. *Medical Journal of Australia*, 157, 651–653.

[8] Hunter, R. W. *et al.* (1993). Can laparoscopic assisted hysterectomy safely replace abdominal hysterectomy? *Western Australia British Journal of Physical Gynaecology*, 100, 932–934.

[9] Jones, R. A. (1993). Laparoscopic hysterectomy; a series of one hundred cases. *Medical Journal of Australia*, 159, 447–449.

[10] Wood, E. C. *et al.* (1994). Laparovaginal hysterectomy. *Australian and New Zealand Journal of Obstetrics and Gynaecology*, 34, 81–84.

[11] Wood, E. C. *et al.* (1994). The replacement of abdominal hysterectomy by the laparovaginal technique—success and limitations. *Australian and New Zealand Journal of Obstetrics and Gynaecology*, 34, 571–574.

[12] The Royal Australian College for Obstetrics and Gynaecology and The Australian Gynaecological Endoscopy Society. (1993). Training in Advanced Operative Laparoscopy. Policy No. 17, 1–3.

[13] Garry, R. *et al.* (1994). Laparoscopic hysterectomy—definition and indications in gynaecological endoscopy; 3, 1–3.

